# The relationship between speed skating coach leadership behavior and athletes’ engagement: the mediating role of athlete psychological fatigue

**DOI:** 10.3389/fpsyg.2025.1690254

**Published:** 2025-12-10

**Authors:** Xu Wang, Qi Feng, Dawei Cao

**Affiliations:** 1 School of Physical Education, Liaoning Normal University, Dalian, China; 2 Graduate Department, Shenyang Sports University, Shenyang, China; 3 School of Physical Education, Huaibei Normal University, Huaibei, China

**Keywords:** coach leadership behavior, athlete engagement, athlete psychological fatigue, speed skating, mediation analysis

## Abstract

**Objective:**

This study aims to explore the association between speed skating coaches’ leadership behavior and athletes’ sports engagement, and to examine the mediating role of athlete psychological fatigue in this relationship.

**Methods:**

A convenience sampling method was adopted to survey 433 speed skating athletes from three provinces in Northeast China. Data were collected using the Coach Leadership Behavior Scale, Athlete Engagement Questionnaire, and Psychological Fatigue in Sports Questionnaire. The obtained data were subjected to descriptive statistical analysis, correlation analysis, regression analysis, and mediation effect testing.

**Results:**

Correlation analysis revealed that authoritarian behavior, a dimension of coach leadership behavior, was significantly negatively correlated with all dimensions of athlete engagement (*p* < 0.01) and significantly positively correlated with all dimensions of athlete psychological fatigue (*p* < 0.01). In contrast, other dimensions of coach leadership behavior (training guidance, democratic, social support, and positive feedback) were significantly positively correlated with all dimensions of athlete engagement (*p* < 0.01) and significantly negatively correlated with all dimensions of athlete psychological fatigue (*p* < 0.01). Additionally, all dimensions of athlete psychological fatigue were significantly negatively correlated with all dimensions of athlete engagement (*p* < 0.01). Regression analysis showed that all dimensions of coach leadership behavior had a significant effect on athlete engagement (*p* < 0.01) and athlete psychological fatigue (*p* < 0.05), while all dimensions of athlete psychological fatigue also had a significant effect on athlete engagement (*p* < 0.05). Mediation effect testing indicated that athlete psychological fatigue played a partial mediating role in the influence of coach leadership behavior on athlete engagement.

**Conclusion:**

This study found significant correlations between coach leadership behavior, athlete psychological fatigue, and athlete engagement, as well as among their respective dimensions. Each dimension of coach leadership behavior could directly influence both athlete engagement and athlete psychological fatigue, while each dimension of athlete psychological fatigue was directly associated with athlete engagement. Athlete psychological fatigue exerted a partial mediating effect in the relationship between coach leadership behavior and athlete engagement.

## 1 Introduction

In the context of increasingly elite competitive sports, maintaining a high level of athlete engagement has become essential for achieving optimal athletic performance (Ma, 2021). Athlete engagement is defined as a positive, persistent, and cognitive-affective experience characterized by confidence, dedication, vigor, and enthusiasm (De Francisco et al., 2018). This psychological state is strongly associated with positive training adaptations and competitive outcomes (Ma, 2021). Conversely, diminished engagement can lead to negative affective states, impaired performance, and ultimately sport dropout. Therefore, identifying factors that enhance athlete engagement is critically important.

The leadership behavior of coaches represents a key environmental factor influencing athletes’ psychological states. Coaches, through specific interpersonal and strategic behaviors, shape the motivational climate and directly affect athletes’ training experiences and competitive outcomes (Lu, 2019). Although previous research has demonstrated a direct association between coaching behavior and athletes’ engagement, the mediating variables involved remain insufficiently explored. This study aims to address this gap by investigating the mediating role of sport psychological fatigue in the relationship between coach leadership behavior and athlete engagement in the context of speed skating. Speed skating was selected due to its high physical and psychological demands, which make athletes particularly susceptible to psychological fatigue, thus providing a relevant context for examining the proposed mediation model. Elucidating this mechanism is expected to provide coaches with evidence-based strategies to mitigate athletes’ psychological fatigue and thereby enhance their engagement.

### 1.1 The relationship between coach leadership behavior and athlete engagement

Coach leadership behavior encompasses a range of actions influenced by the coach’s personal attributes, managerial skills, and situational factors related to the team and individual athletes. Through these behaviors, coaches guide athletes toward achieving collective and individual goals (Lu, 2019). Athlete engagement, conceptualized as a positive and persistent state of mind characterized by confidence, dedication, vigor, and enthusiasm, is a direct precursor to performance in training and competition (De Francisco et al., 2018).

Empirical evidence supports a positive association between coach-created motivational climates and athlete engagement. For instance, a perceived mastery-oriented climate fostered by coaches has been shown to promote engagement (Curran et al., 2015). Similarly, coaches’ caring behaviors have been found to significantly enhance athletes’ role engagement, a construct closely aligned with athlete engagement (Shi et al., 2017). Furthermore, research indicates that both global and specific dimensions of coach leadership behavior are significant predictors of engagement, albeit through distinct pathways (Yuanyuan et al., 2021). Longitudinal designs provide stronger evidence for causality; a study of amateur basketball players demonstrated that coaches’ caring behavior not only correlated with but also predicted athlete engagement levels 12 weeks later (Qianqian et al., 2022). Collectively, these findings underscore the critical role of adaptive coach leadership behaviors in fostering athlete engagement.

Based on this, this study proposes research hypothesis

*H1*: Speed skating coaches’ leadership behavior will be positively associated with athlete engagement.

### 1.2 The relationship between coaching leadership behavior and athlete psychological fatigue

Sport psychological fatigue is a syndrome comprising emotional/physical exhaustion, sport devaluation, and a reduced sense of accomplishment (Raedeke, 1997). Coaching behaviors are significant antecedents to this condition. Certain maladaptive behaviors, such as excessive autocratic coaching and an overemphasis on punitive training feedback, have been identified as risk factors, predictive of athletes’ exhaustion and devaluation (Altahayneh, 2013). Conversely, supportive coaching behaviors serve a protective function. Social support from coaches has been directly linked to lower levels of psychological fatigue (Raedeke and Smith, 2004), whereas a lack of timely support can exacerbate it (Defreese and Smith, 2014). Importantly, coaches’ autonomy-supportive behaviors not only facilitate positive states but also actively buffer against the development of psychological fatigue (Yue et al., 2023; Zhengmao and Jian, 2017). This protective effect extends to the relational level, where a high-quality coach-athlete relationship is a significant negative predictor of athletes’ psychological fatigue (Zhengmao et al., 2021). It can be seen that establishing a harmonious ‘coach-athlete’ relationship is an important protective factor against psychological fatigue.

Based on this, this study proposes research hypothesis

*H2*: Speed skating coaches’ leadership behavior will be negatively associated with athletes’ sport psychological fatigue.

### 1.3 The relationship between athlete psychological fatigue and athlete engagement

A robust negative relationship exists between sport psychological fatigue and athlete engagement. Theoretical models, such as the Sport Commitment Model, posit that lower engagement can predispose athletes to burnout (Scanlan et al., 1993; Schmidt and Stein, 2010). Empirical evidence confirms that the onset of psychological fatigue directly undermines engagement, likely through shared antecedents like chronic stress. Stress has been shown to negatively impact engagement (Kamimura et al., 2018) and is a well-established precursor to burnout (Smith, 1986). Furthermore, athlete engagement itself is a resilience factor that promotes mental health and mitigates negative states, including psychological fatigue (Lu et al., 2016). A recent mediation study illustrated that a positive coach-athlete relationship reduces psychological fatigue precisely by first enhancing athlete engagement, highlighting engagement’s central role in this dynamic (Zhengmao et al., 2021).

Based on this evidence, this study proposes research hypothesis

*H3*: Sport psychological fatigue will be negatively associated with athlete engagement among speed skaters.

### 1.4 The proposed mediated model

The reviewed literature suggests that coach leadership behavior influences athlete engagement both directly and indirectly by affecting athletes’ levels of psychological fatigue. To integrate these pathways, we propose a mediation model and a fourth hypothesis

*H4*: Sport psychological fatigue will mediate the relationship between coach leadership behavior and athlete engagement.

**Figure 1:**
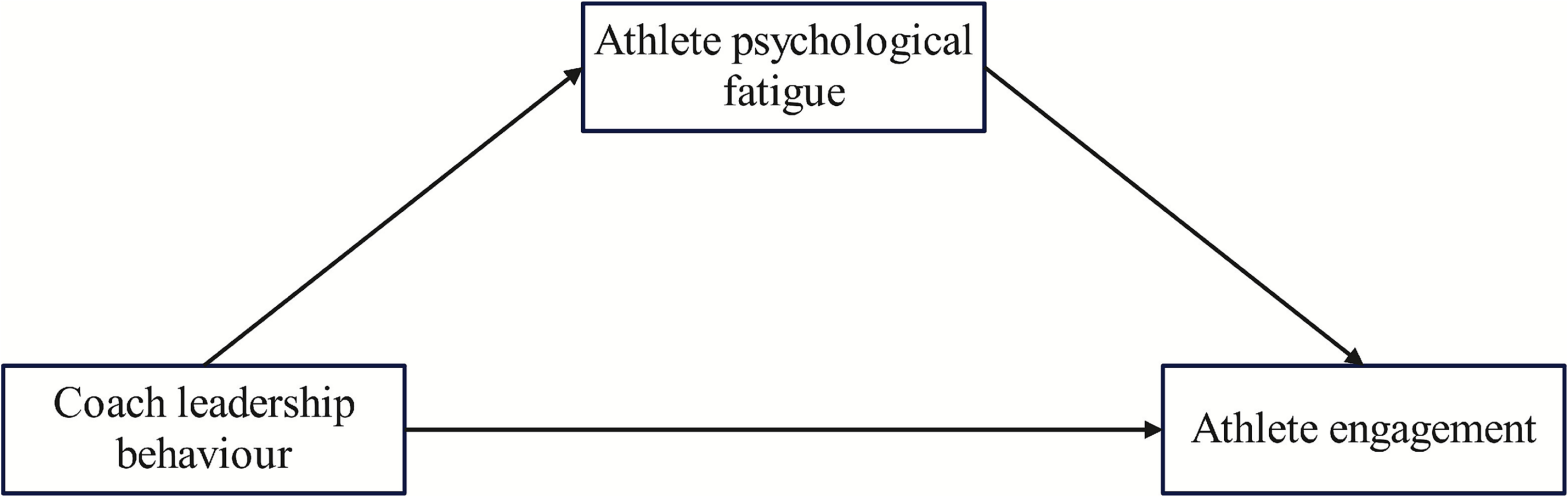
Hypothetical model diagram.

In summary, this study proposes and tests a theoretical model (Figure 1) comprising four hypotheses to examine the direct and indirect effects of coach leadership behavior on athlete engagement via sport psychological fatigue in speed skaters.

*H1*: Speed skating coaches’ leadership behavior will be positively associated with athlete engagement.

*H2*: Speed skating coaches’ leadership behavior will be negatively associated with sport psychological fatigue.

*H3*: Sport psychological fatigue will be negatively associated with athlete engagement.

*H4*: Sport psychological fatigue will mediate the relationship between coach leadership behavior and athlete engagement.

## 2 Methods

### 2.1 Participants and procedures

From January to March 2024, a convenience sampling method was adopted, and an online questionnaire was used to recruit competitive speed skaters from the Winter Sports Management Centers in Liaoning, Jilin, and Heilongjiang provinces of China. The three northeastern provinces are also the main concentrated regions for the development of ice and snow sports in China, with nearly 90% of China’s speed skaters coming from these three provinces. This approach was taken to ensure the representativeness of the sample as much as possible.

In total, 433 valid questionnaires were collected and included in the final analysis. The demographic and athletic characteristics of the participant sample are summarized in Table 1.

**Table 1:** Characteristics of speed skaters (*N* = 433).

Variable		Number	Percentage (%)
Gender	Male	237	54.73%
	Female	196	45.27%
Age group	16–19 years old	132	30.48%
	20–23 years old	195	45.04%
	24–27 years old	106	24.48%
Level of athleticism	Second-level athlete	158	36.49%
	First-level athlete	162	37.41%
	Elite athlete	113	26.10%
Number of years training	1–7 years	127	29.33%
	8–12 years	189	43.65%
	13–18 years	117	27.02%

Athlete levels are defined according to the Chinese National Sports General Administration’s grading system. “Second-level athlete” corresponds to regional competitive level, “First-level athlete” to national level, and “Elite athlete” to international or elite level.

### 2.2 Measuring tools

#### 2.2.1 Coaching leadership behavior

The Chinese version of the Coaching Leadership Behavior Scale, revised by Duanwei (2016), was used to assess coaches’ leadership behaviors. This instrument contains 31 items across five dimensions: Training Guidance Behavior, Democratic Behavior, Authoritarian Behavior, Social Support Behavior, and Positive Feedback Behavior. Each item was rated on a 5-point Likert scale. In the present sample, the internal consistency reliabilities (Cronbach’s *α*) for the subscales ranged from 0.898 to 0.941. Confirmatory Factor Analysis (CFA) indicated good structural validity (*χ*^2^/df = 1.756, CFI = 0.973, TLI = 0.970, RMSEA = 0.042, RMR = 0.066), supporting the scale’s reliability for this sample.

#### 2.2.2 Athlete engagement

Athlete engagement was measured using the Athlete Engagement Questionnaire, adapted into Chinese by Lu (2014). The scale comprises 16 items grouped into four dimensions: Confidence, Vigor, Dedication, and Enthusiasm. Responses were collected on a 5-point Likert scale ranging from 1 (“never”) to 5 (“always”). In this study, Cronbach’s α values for the subscales ranged from 0.875 to 0.891. Confirmatory Factor Analysis (CFA) indicated good structural validity (*χ*^2^/df = 2.456, CFI = 0.967, TLI = 0.959, RMSEA = 0.058, RMR = 0.061), supporting the scales’ reliability for this sample.

#### 2.2.3 Athlete psychological fatigue

The Chinese adaptation of the Sport Psychological Fatigue Questionnaire, developed by Liwei and Zhixiong (2004), was administered to assess psychological fatigue. This 15-item instrument consists of three subscales (Reduced Sense of Accomplishment, Emotional/Physical Exhaustion, and Sport Devaluation) and employs a 5-point Likert response format from 1 (“never”) to 5 (“always”). For the current sample, subscale reliabilities ranged between 0.868 and 0.881. Confirmatory Factor Analysis (CFA) indicated good structural validity (*χ*^2^/df = 1.316, CFI = 0.992, TLI = 0.990, RMSEA = 0.027, RMR = 0.034), supporting the scales’ reliability for this sample.

### 2.3 Data processing

All questionnaire data were initially compiled using Excel 2021. Subsequent statistical analyses were conducted with SPSS 26.0 and Amos 24.0. Reliability analysis, descriptive statistics, and correlation analyses were performed using SPSS. Common method bias was assessed using Harman’s single-factor test. Pearson correlation analysis was employed to examine bivariate relationships among the key variables. A comprehensive structural equation model (SEM) was constructed in Amos 24.0 to test the mediating role of psychological fatigue, incorporating all five dimensions of leadership behavior and controlling for gender, years of training, and competition level. The significance of indirect effects was evaluated using a bootstrap approach with 5,000 resamples. Mediation was considered statistically significant if the 95% bias-corrected bootstrap confidence interval did not include zero.

## 3 Results

### 3.1 Common method bias test

To mitigate potential common method bias, anonymous survey administration and reverse-scored items were employed during data collection. Furthermore, Harman’s single-factor test was conducted (Hao and Lirong, 2004). The results indicated the presence of 12 factors with eigenvalues exceeding 1. The variance explained by the first factor was 30.01%, which is below the critical threshold of 40% (Dandan and Zhonglin, 2020), suggesting that common method bias was not a substantial concern in this study.

### 3.2 Correlation analysis

Correlation analysis revealed that authoritarian coaching behavior was significantly negatively correlated with all dimensions of athlete engagement (*p* < 0.01) and significantly positively correlated with all dimensions of sport psychological fatigue (*p* < 0.01). In contrast, all other dimensions of coaching leadership behavior (training guidance, democratic, social support, and positive feedback behaviors) were significantly positively correlated with all dimensions of athlete engagement (*p* < 0.01) and significantly negatively correlated with all dimensions of sport psychological fatigue (*p* < 0.01). Additionally, all dimensions of sport psychological fatigue were significantly negatively correlated with all dimensions of athlete engagement (*p* < 0.01). High intercorrelations among some subscales (e.g., >0.70) suggested potential multicollinearity, which was further examined in regression models using variance inflation factors (VIF). All VIF values were below 10, indicating that multicollinearity was not a critical issue. Detailed correlation coefficients are presented in Table 2.

**Table 2:** Descriptive statistics and correlation analysis of each variable (*N* = 433).

Variables	M	SD	1	1.1	1.2	1.3	1.4	1.5	2	2.1	2.2	2.3	2.4	3	3.1	3.2	3.3
1 Coaching leadership behavior	3.21	0.61	1														
1.1 Training guidance behavior	3.29	1.10	0.726**	1													
1.2 Democratic behavior	3.29	1.03	0.763**	0.398**	1												
1.3 Positive feedback behavior	3.30	1.03	0.729**	0.416**	0.425**	1											
1.4 Social support behavior	3.34	1.08	0.725**	0.407**	0.454**	0.438**	1										
1.5 Authoritarian behavior	2.64	1.08	−0.350**	−0.405**	−0.421**	−0.421**	−0.375**	1									
2 Athlete engagement	3.59	0.78	0.496**	0.380**	0.399**	0.429**	0.404**	−0.393**	1								
2.1 Confidence	3.62	0.98	0.330**	0.268**	0.241**	0.305**	0.281**	−0.287**	0.772**	1							
2.2 Vigor	3.57	0.97	0.392**	0.253**	0.342**	0.321**	0.323**	−0.260**	0.788**	0.459**	1						
2.3 Dedication	3.58	1.01	0.455**	0.349**	0.379**	0.366**	0.358**	−0.325**	0.806**	0.467**	0.534**	1					
2.4 Enthusiasm	3.59	0.96	0.395**	0.339**	0.304**	0.370**	0.320**	−0.381**	0.811**	0.526**	0.517**	0.546**	1				
3 Athlete psychological fatigue	3.74	0.72	−0.466**	−0.355**	−0.366**	−0.416**	−0.368**	0.354**	−0.370**	−0.290**	−0.271**	−0.291**	−0.323**	1			
3.1 Reduced Sense of Accomplishment	3.76	0.87	−0.381**	−0.296**	−0.283**	−0.334**	−0.319**	0.284**	−0.327**	−0.259**	−0.261**	−0.268**	−0.252**	0.849**	1		
3.2 Emotional/physical exhaustion	3.70	0.82	−0.412**	−0.319**	−0.331**	−0.371**	−0.318**	0.327**	−0.305**	−0.234**	−0.208**	−0.222**	−0.307**	0.846**	0.567**	1	
3.3 Sport Devaluation	3.74	0.86	−0.400**	−0.297**	−0.324**	−0.359**	−0.307**	0.296**	−0.313**	−0.248**	−0.223**	−0.254**	−0.270**	0.865**	0.597**	0.613**	1

**p < 0.01.

### 3.3 Regression analysis

To further examine the complex relationships among coaching leadership behavior, athlete engagement, and sport psychological fatigue, a series of regression analyses were conducted using three distinct regression models, controlling for gender, years of training, and competition level.

#### 3.3.1 Regression analysis of coach leadership behavior on athlete engagement

A regression model was constructed with the five dimensions of coaching leadership behavior as independent variables and athlete engagement as the dependent variable. The results indicated that training guidance behavior, democratic behavior, positive feedback behavior, and social support behavior significantly positively predicted athlete engagement, whereas authoritarian behavior significantly negatively predicted athlete engagement. Complete results are provided in Table 3.

**Table 3:** Regression analysis of coach leadership behavior on athlete engagement (*N* = 433).

	Non-standardised coefficient		Standardised coefficient	*t*	*p*	Collinearity diagnosis	
	*B*	S.E.	Beta			VIF	Tolerance
Constant	2.401	0.221	-	10.878	0.000**	–	–
Training guidance behavior	0.089	0.034	0.125	2.599	0.010**	1.413	0.708
Democratic behavior	0.103	0.037	0.137	2.780	0.006**	1.485	0.674
Positive feedback behavior	0.144	0.037	0.190	3.861	0.000**	1.480	0.676
Social support behavior	0.109	0.035	0.152	3.109	0.002**	1.468	0.681
Authoritarian behavior	−0.107	0.035	−0.148	−3.084	0.002**	1.415	0.707
*R* ^2^	0.303						
Adjusted *R*^2^	0.295						
*F*	*F*(5.427) = 37.105**						
D-W value	1.724						

Dependent variable: athlete engagement *p < 0.05, **p < 0.01.

#### 3.3.2 Regression analysis of coach leadership behavior on athlete psychological fatigue

Using the dimensions of coaching leadership behavior as independent variables and sport psychological fatigue as the dependent variable, regression analysis showed that training guidance, democratic, positive feedback, and social support behaviors significantly negatively predicted sport psychological fatigue. Conversely, authoritarian behavior significantly positively predicted sport psychological fatigue. See Table 4 for detailed results.

**Table 4:** Regression analysis of coach leadership behavior on athlete psychological fatigue (*N* = 433).

	Non-standardised coefficient		Standardised coefficient	*t*	*p*	Collinearity diagnosis	
	*B*	S.E.	Beta			VIF	Tolerance
Constant	4.837	0.211	-	22.963	0.000**	–	–
Training guidance behavior	−0.079	0.033	−0.120	−2.434	0.015*	1.413	0.708
Democratic behavior	−0.085	0.035	−0.121	−2.393	0.017*	1.485	0.674
Positive feedback behavior	−0.146	0.036	−0.208	−4.111	0.000**	1.480	0.676
Social support behavior	−0.086	0.034	−0.129	−2.564	0.011*	1.468	0.681
Authoritarian behavior	0.079	0.033	0.118	2.387	0.017*	1.415	0.707
R^2^	0.263						
Adjusted R^2^	0.254						
F	F(5.427) = 30.421**						
D-W value	1.100						

Dependent variable: athlete psychological fatigue *p < 0.05, **p < 0.01.

#### 3.3.3 Regression analysis of athlete psychological fatigue on athlete engagement

When the dimensions of sport psychological fatigue were entered as independent variables and athlete engagement as the dependent variable, results indicated that reduced sense of accomplishment, emotional/physical exhaustion, and sport devaluation all significantly negatively predicted athlete engagement. Full results are presented in Table 5.

**Table 5:** Regression analysis of athlete psychological fatigue on athlete engagement (*N* = 433).

	Non-standardised coefficient		Standardised coefficient	*t*	*p*	Collinearity diagnosis	
	*B*	S.E.	Beta			VIF	Tolerance
Constant	5.076	0.184	–	27.561	0.000**	–	–
Reduced sense of accomplishment	−0.161	0.053	−0.180	−3.047	0.002**	1.728	0.579
Emotional/physical exhaustion	−0.117	0.057	−0.123	−2.062	0.040*	1.782	0.561
Sport devaluation	−0.118	0.056	−0.130	−2.116	0.035**	1.878	0.533
*R* ^2^	0.137						
Adjusted *R*^2^	0.131						
*F*	*F*(3.429) = 22.727**						
D-W value	1.551						

Dependent variable: athlete engagement *p < 0.05, **p < 0.01.

### 3.4 Mediating effect test

A comprehensive structural equation model (SEM) was tested using Amos 24.0, incorporating all five leadership dimensions and controlling for gender, years of training, and competition level (Zhonglin and Baojuan, 2014). The bootstrap method with 5,000 resamples was applied (Preacher and Hayes, 2004). All variables were standardized prior to analysis to facilitate interpretation and comparison. Coaching leadership behavior dimensions were treated as independent variables, athlete engagement as the dependent variable, and sport psychological fatigue as the mediating variable.

The results of the mediation analysis are summarized in Table 6. The direct, indirect, and total effects for each leadership dimension are reported.

**Table 6:** Testing the mediating effect of athlete psychological fatigue.

	Mediation effect	Effect category	Effect value	Proportion of total effect	Boot standard error	Bootstrap lower	Bootstrap upper
Model 1	Training guidance behavior → athlete psychological fatigue → athlete engagement	Direct effect	0.202**	74.93%	0.033	0.138	0.266
		Indirect effect	0.068**	25.07%	0.068	0.045	0.096
		Total effect	0.270**		0.032	0.208	0.332
Model 2	Democratic behavior → athlete psychological fatigue → athlete engagement	Direct effect	0.229**	76.31%	0.035	0.162	0.297
		Indirect effect	0.071**	23.69%	0.013	0.047	0.099
		Total effect	0.301**		0.033	0.235	0.366
Model 3	Authoritarian behavior → athlete psychological fatigue → athlete engagement	Direct effect	−0.217**	76.31%	0.033	−0.282	−0.152
		Indirect effect	−0.067**	23.69%	0.012	−0.092	−0.046
		Total effect	−0.284**		0.032	−0.347	−0.221
Model 4	Social support behavior → athlete psychological fatigue → athlete engagement	Direct effect	0.223**	76.70%	0.033	0.158	0.287
		Indirect effect	0.068**	23.30%	0.012	0.045	0.093
		Total effect	0.290**		0.032	0.228	0.352
Model 5	Positive feedback behavior → athlete psychological fatigue → athlete engagement	Direct effect	0.253**	77.60%	0.035	0.183	0.322
		Indirect effect	0.073**	22.40%	0.015	0.045	0.105
		Total effect	0.326**		0.033	0.261	0.391

*p < 0.05, **p < 0.01.

**Figure 2:**
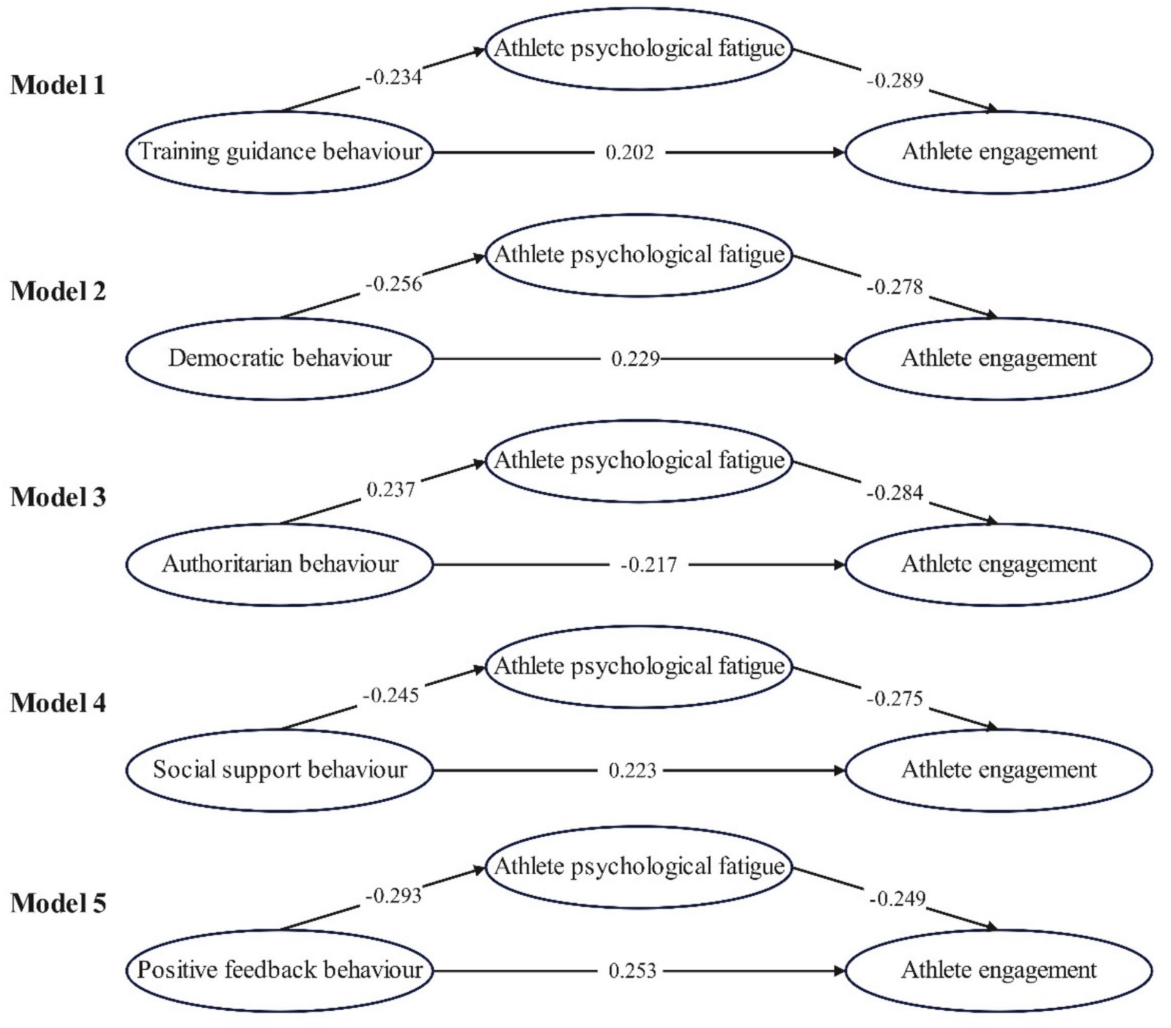
Mediator model diagram.

As none of the bootstrap confidence intervals for the indirect effects included zero, the mediating role of sport psychological fatigue was statistically significant in all models (see Figure 2).

## 4 Discussion

### 4.1 The associations of coach leadership behavior with athlete engagement

The findings confirm that coaching leadership behavior significantly influences athlete engagement, supporting Hypothesis H1. Specifically, all dimensions of leadership behavior—except authoritarian behavior—demonstrated a significant positive correlation with athlete engagement, suggesting that constructive coaching practices effectively enhance engagement. In contrast, authoritarian behavior correlated negatively with all engagement dimensions, indicating its detrimental association. These results align with previous research (Yuanyuan et al., 2021).

As central figures in athletic development, coaches exert considerable influence over athletes’ daily routines, training, and competitive experiences. Positive leadership behaviors, such as providing support, encouragement, and autonomy, can foster athletes’ enthusiasm and sustained involvement. Conversely, authoritarian approaches may undermine athletes’ psychological well-being and diminish engagement. Meeting athletes’ psychological needs is critical to sustaining motivation and effort in training and competition (Yuanyuan et al., 2021), and higher engagement in turn provides a foundation for achieving performance goals (Lu et al., 2016).

### 4.2 The associations of coach leadership behavior with athlete psychological fatigue

The results indicate that coaching leadership behavior is directly associated with sport psychological fatigue, supporting Hypothesis H2. All leadership dimensions except authoritarian behavior negatively predicted psychological fatigue, indicating their protective role. Authoritarian behavior, however, showed a positive relationship with psychological fatigue, consistent with earlier studies (Defreese and Smith, 2014). Prior research indicates that coaches’ social support helps mitigate psychological fatigue (Raedeke and Smith, 2004), whereas cold or controlling behaviors can exacerbate it (Defreese and Smith, 2014).

Athletes operate in a hierarchical environment where coaches play a decisive role in their mental and performance outcomes. Behaviors that convey respect, provide encouragement, and grant autonomy help prevent psychological fatigue by strengthening self-efficacy and promoting positive affect (Yue et al., 2023). In contrast, authoritarian tactics may increase pressure and negative emotions, thereby elevating fatigue. A supportive coaching approach helps athletes feel valued, thereby enhancing their psychological resilience and reducing the impact of negative emotional states.

### 4.3 The associations of athlete psychological fatigue with athlete engagement

This study also confirms that sport psychological fatigue is directly associated with reduced athlete engagement, supporting Hypothesis H3. This result is consistent with the sport commitment model (Scanlan et al., 1993), which posits that engagement and burnout are interrelated (Schmidt and Stein, 2010). When athletes experience psychological fatigue, they struggle to fully invest in training and competition, which impairs performance and mental health. Therefore, effective interventions aimed at reducing psychological fatigue are essential for maintaining engagement.

Notably, athlete engagement may also serve as a buffer against negative psychological states (Lu et al., 2016), suggesting a bidirectional relationship between engagement and fatigue. Thus, coaches should prioritize both fostering engagement and mitigating fatigue to support athletes’ holistic well-being.

### 4.4 The mediating role of athlete psychological fatigue

Mediation analyses revealed that sport psychological fatigue partially mediates the relationship between each dimension of coaching leadership behavior and athlete engagement, supporting Hypothesis H4. These results underscore the importance of addressing psychological fatigue to improve athlete engagement. For instance, effective training guidance not only directly increased engagement but also reduced psychological fatigue, which further enhanced engagement. This indicates that coaches should integrate psychological monitoring and support into their training strategies to sustain athlete involvement.

Similarly, democratic behavior promoted engagement both directly and indirectly by alleviating fatigue. By fostering autonomy and involving athletes in decision-making, coaches can enhance motivation and reduce pressure (Yuanyuan et al., 2021), thereby supporting mental freshness and sustained engagement. Authoritarian behavior increased psychological fatigue, which in turn reduced engagement. This style creates pressure and emotional strain, impairing athletes’ focus and motivation while damaging coach–athlete relationships (Dong et al., 2022). In such environments, athletes are more likely to experience fatigue and disengagement.

Social support and positive feedback behaviors also reduced fatigue and enhanced engagement. Supportive interactions help athletes manage stress and build positive relationships (Jun, 2018), thereby strengthening their psychological readiness and commitment. Positive feedback validates effort and achievement, boosting self-confidence and drive (Qiang et al., 2018), which further sustains engagement and performance.

### 4.5 Limitations and prospects

Several limitations should be noted. First, the cross-sectional design precludes causal inference. Future studies should adopt longitudinal or experimental designs to establish temporal precedence and causality. Second, the current set of variables is limited; incorporating additional factors such as psychological resilience, training satisfaction, and team efficacy could provide a more comprehensive model. Third, this study employed convenience sampling to conduct investigations across three provinces in Northeast China. The sample may not fully represent speed skaters of all backgrounds and levels nationwide. The use of convenience sampling may limit the generalizability of the findings to the broader population of speed skaters. It is recommended that subsequent research enhance the external validity of conclusions by adopting more comprehensive sampling methods and incorporating diverse samples. Finally, this study focused solely on speed skating, future research should examine whether these relationships hold across different sports and cultural contexts.

## 5 Conclusion

This study demonstrates that authoritarian coaching behavior is negatively correlated with athlete engagement and positively correlates with sport psychological fatigue. All other leadership dimensions are positively correlated with engagement and negatively with fatigue. Sport psychological fatigue is negatively correlated with engagement and mediates the relationship between coaching behavior and athlete engagement. These findings highlight the importance of positive coaching practices and psychological support in fostering athlete engagement and well-being.

## Data Availability

The original contributions presented in the study are included in the article/Supplementary material, further inquiries can be directed to the corresponding author.
